# Circulating Soluble ST2 Predicts All-Cause Mortality in Severe Heart Failure Patients with an Implantable Cardioverter Defibrillator

**DOI:** 10.1155/2020/4375651

**Published:** 2020-11-17

**Authors:** Zhi-wei Hou, Hai-bo Yu, Yan-chun Liang, Yang Gao, Guo-qing Xu, Min Wu, Zhu Mei, Zu-lu Wang, Zhi-guo Li, Yu-ying Li, Hai-xu Song, Jia-yin Li, Ya-ling Han

**Affiliations:** ^1^Department of Cardiology, Cardiovascular Research Institute, General Hospital of Northern Theater Command, Shenyang 110016, China; ^2^Huazhong University of Science and Technology, Wuhan 430030, China; ^3^Air Force Medical University, Xian 710000, China; ^4^Northeastern University, Shenyang 110016, China

## Abstract

**Background:**

Heart failure (HF) is the terminal stage of all cardiovascular events. Although implantable cardioverter defibrillator (ICD) therapies have reduced mortality among the high-risk HF population, it is necessary to determine whether certain factors can predict mortality even after cardiac device implantation. Growth stimulation expressed gene 2 (ST2) is an emerging biomarker for HF patient stratification in different clinical settings.

**Aims:**

This study aimed to investigate the relationship between baseline soluble ST2 (sST2) levels in serum and the clinical outcomes of high-risk HF patients with device implantation.

**Methods:**

Between January 2017 and August 2018, we prospectively recruited consecutive patients implanted with an ICD for heart failure, with LVEF ≤35% as recommended, and analyzed the basic characteristics, baseline serum sST2, and NT-proBNP levels, with at least 1-year follow-up. All-cause mortality was the primary endpoint.

**Results:**

During a 643-day follow-up, all-cause mortality occurred in 16 of 150 patients (10.67%). Incidence of all-cause mortality increased significantly in patients with sST2 levels above 34.98846 ng/ml (16.00% *vs*. 5.33%, *P* = 0.034). After adjusting the model (age, gender, device implantation, prevention of sudden death, LVEDD, LVEF, WBC and CLBBB, hsTNT, etiology, and eGFR) and the model combined with NT-proBNP, the risk of all-cause death was increased by 2.5% and 1.9%, respectively, per ng/ml of sST2. The best sST2 cutoff for predicting all-cause death was 43.42671 ng/ml (area under the curve: 0.72, sensitive: 0.69, and specificity: 0.69). Compared to patients with sST2 levels below 43.42671 ng/ml, the risk of all-cause mortality was higher in those with values above the threshold (5.1% vs. 21.2%, *P* = 0.002). ST2 level ≥43.42671 ng/ml was an independent predictor of all-cause mortality (HR: 3.30 [95% CI 1.02–10.67]). Age (HR: 1.06 [95% CI: 1.01–1.12]) and increased NT-proBNP per 100 (HR: 1.02 [95% CI: 1.01–1.03]) were also associated with all-cause mortality in ICD patients.

**Conclusions:**

sST2 level was associated with risk of all-cause mortality, and a threshold of 43.43 ng/ml showed good distinguishing performance to predict all-cause mortality in patients with severe heart failure, recommended for ICD implantation. Patients with sST2 levels more than 43.42671 ng/ml even after ICD implantation should therefore be monitored carefully.

## 1. Introduction

Heart failure (HF) is the terminal stage of all cardiovascular events and is a major cause of mortality in developing and developed countries [1, 2]. Although implantable cardioverter defibrillator (ICD) therapies among the high-risk HF population have reduced the mortality, factors that can predict mortality even after implantation of cardiac devices need to be identified [3, 4]. Plasma biomarkers may be an effective tool for monitoring the prognosis for the risk of sudden cardiac death (SCD) in high-risk HF patients. However, beyond natriuretic peptides (NP), use of biomarkers for risk assessment is still being debated [5–8]. There are some risks for the confounding effects of brain natriuretic peptide (BNP) or N-terminal probrain natriuretic peptide (NT-proBNP) such as in renal insufficiency patients, in whom BNP may be elevated. Moreover, high concentrations of BNP imply great differences in values according to changes in clinical situations [9–11].

Recently, growth stimulation expressed gene 2 (ST2) is a novel emerging biomarker for patient stratification in different clinical settings [12–14]. ST2 is a member of the Toll-interleukin 1 (IL-1) receptor superfamily that includes two unique proteins: truncated circulating soluble ST2 (sST2) and a membrane-bound form (lST2). sST2 competes with lST2 to bind with interleukin-33 (IL-33), which is involved in ameliorating myocardial hypertrophy and fibrosis in response to cardiovascular stretch [15–18]. Originally, elevated levels of sST2 were reported to be associated with the severity of adverse cardiac remodeling and tissue fibrosis in HF patients. Further, increased levels of sST2 are correlated with more severe clinical symptoms and with other objective measures of HF severity such as higher C-reactive protein, higher natriuretic peptide levels, lower left ventricular ejection fraction (LVEF), and higher diastolic filling pressure [12, 14, 19–23]. Using a nested case-control analysis of patients with ambulatory HF, Bayes-Genis and Giuseppe et al. showed a significant correlation between elevated sST2 concentrations and risk of SCD [13, 24]. In a recent study, Hicham et al. also provided data on a biomarker substudy from the MADIT-CRT trial. They reported that both baseline and serial elevated sST2 levels could predict an increasing risk of death, HF, or ventricular arrhythmic events [25]. Meanwhile, the prognostic value of sST2 has been confirmed in acute dyspnea, acute coronary syndrome, and acute and chronic HFrEF, but it has been less studied in HF patients with device implantation, especially in patients with EF no more than 35% [26, 27]. However, the use of sST2 level to predict cardiac MACE was not clear in HF patients after ICD implantation.

This study aimed to investigate the relationship between baseline sST2 levels in serum and the clinical outcomes of high-risk HF patients after ICD implantation and to find a suitable sST2 level that could evaluate the mortality risk of HF patients even after ICD therapy.

## 2. Methods

### 2.1. Patient Population

Between January 2017 and August 2018, patients with chronic heart failure were prospectively enrolled in our study at the cardiology department of the General Hospital of Northern Theater Command. Patients aged more than 18 years, with LVEF ≤35%, and recommended and implanted with an ICD as per the guidelines, were considered eligible. The exclusion criteria were age <18 years, acute coronary syndrome, severe heart valve disease, severe liver and kidney disease, severe anemia, end-stage chronic obstructive pulmonary disease, acute asthma attack, trauma or surgery of any type within the preceding 8 weeks, mental disorders, rheumatoid arthritis, and systemic lupus erythematosus. All the patients were followed up for at least 1 year; the final follow-up point was September 30, 2019. All-cause mortality was the primary endpoint of this study. Patient management was at the discretion of the treating physician and was provided in accordance with the recommended guidelines.

Blood was drawn from a forearm vein and was collected in an ethylenediaminetetraacetic acid (EDTA) tube before device implantation when patients' heart function was relatively stable. Samples were centrifuged, and plasma was frozen within 1 h of sampling at −80°C for pending analysis.

### 2.2. Biomarker Analysis

We analyzed two plasma biomarkers that reflect a range of pathophysiological processes in CHF:

1. Serum ST2, the soluble form of ST2 (sST2), a novel marker of myocardial stretch
2. NT-proBNP, an established marker of myocardial stretch

These biomarkers were chosen based on previous studies in patients with CHF that demonstrated their independent association with mortality and SCD.

Serum samples and plasma samples were transported under controlled conditions to a central laboratory for batch analysis of sST2 and NT-proBNP levels. sST2 concentrations were determined in serum by single measurement using a quantitative sandwich monoclonal enzyme-linked immunosorbent assay (Presage ST2 Assay, Critical Diagnostics, Inc., San Diego, California). In our hands, the average intra-assay coefficient of variation was 5.0%, in line with the average interassay coefficient of variation of 5.2%, as reported by the manufacturer. NT-proBNP concentrations were determined in plasma using the Elecsys NT-proBNP electrochemiluminescent sandwich immunoassay on a Cobas 8000 analyzer (Roche Diagnostics, Ltd., Rotkreuz, Switzerland). Analysts were blinded to the patients' characteristics and endpoints.

### 2.3. Medical Therapy and Clinical Follow-Up

Treatment for heart failure with evidence-based medical therapies such as beta-blockers, angiotensin-converting enzyme inhibitors (ACEIs) or angiotensin receptor blockers (ARBs), and aldosterone receptor antagonists was optimized in all patients. Device programming was at the discretion of the treating physician. Following enrollment, patients were followed up for 2–4 days on a monthly basis, with a hospital visit or via a remote patient management system. Patients under remote follow-up also attended the hospital every 3 months. At each follow-up, the device was interrogated. The occurrence of any ICD and CRT-D therapy was recorded, and survival was recorded. Clinical information regarding all-cause mortality and major adverse cardiac events (MACE) including cardiac death and requirement for heart transplantation was gathered during a follow-up of 643 days after enrollment. The information was collected by the treating cardiologists who were unaware of the patients' biomarker levels. The cause of death was ascertained by reviewing medical records and contacting the patients/family members. Causes of death were categorized according to a modified Hinkle–Thaler classification [28].

The study complied with the Declaration of Helsinki and was approved by the local research ethics committee. Written informed consent was obtained from all patients.

### 2.4. Statistical Analysis

Continuous variables are presented as mean ± SD or medians (interquartile ranges [IQR]), and categorical variables are presented as counts and percentages. Normal distribution continuous variables were compared by Student's *t*-test, and non-normal distribution data were compared using the Wilcoxon rank sum test. Categorical variables were compared using the chi-square test or Fisher's exact test. Cumulative event rates for clinical outcomes between groups were calculated based on Kaplan–Meier estimates. Survival curves for time-to-event variables were compared using the log-rank test. A receiver operating characteristic curve for serum sST2 was constructed to assess its predictive accuracy for all-cause mortality. The area under the curve for SRI was computed to identify the Youden index (best cutoff) for all-cause death. Multivariable Cox proportional hazards models were constructed to identify independent predictors of all-cause mortality. The variables in the multivariable analysis included age, gender, device implantation, prevention of sudden death, left ventricular end-diastolic diameter (LVEDD), left ventricular ejection fraction (LVEF), white blood cell (WBC), left bundle branch block (LBBB), etiology, estimated glomerular filtration rate (eGFR), and N-terminal probrain natriuretic peptide (NT-proBNP). Statistical analyses were performed using SPSS version 22.0 (SPSS, Chicago, Illinois), and *P* values <0.05 were considered to indicate statistically significant differences.

## 3. Results

### 3.1. Baseline Characteristics

At the baseline, 150 patients were available for the analysis of sST2. Of these, 113 patients (75.33%) were men, and the mean age was 61.63 ± 11.14 years (range 25–86 years). Before device placement, basic LVEF was 29.41 ± 4.69% ranging from 19% to 35%. Of all patients, most were in the New York Heart Association functional class (NYHA FC) III and IV (103 patients, 68.67%). In all, 80 (53.33%) patients were implanted with ICD (single-ICD or dual-ICD), and the other 70 (46.67%) patients were implanted with CRT-D. Of all patients, 70 (46.67%) patients were diagnosed with ischemic heart failure, 110 (73.33%) with primary prevention for sudden cardiac death, and 62 (41.33%) with complete left bundle branch block (CLBBB). The average sST2 concentration was 41.22 ± 24.61 ng/ml with levels ranging from 12.81 to 204.78 ng/ml. Overall, we divided the population into two groups according to the median sST2 level (ST2 ≥34.9885). Baseline characteristics for both groups are shown in Table 1. The median level of sST2 concentration was 47.19 (42.24–62.94) ng/ml in the high sST2 level group, whereas it was 26.20 (22.18–31.10) ng/ml in the low sST2 group.

### 3.2. Follow-Up

During a 643-day follow-up (interquartile interval: 442 d–766 d), all-cause mortality occurred in 16 patients (10.67%) among the 150 patients. The cause of death was classified as cardiovascular death in 14 patients (9.33%). Among the 14 patients, 9 patients (64.28%) died of terminal heart failure, and 5 patients (35.72%) died of arrhythmia or sudden cardiac death. The median level of sST2 concentration was 45.98 (35.09–67.29) in the patients who died, whereas it was 33.80 (25.72–45.43) in the surviving group(*P* = 0.0046). Patients who died were older and had significantly higher levels of hsTNT and NT-proBNP and lower levels of eGFR than patients who survived during follow-up (Table S1).

### 3.3. Prediction of All-Cause Mortality

**Figure 1:**
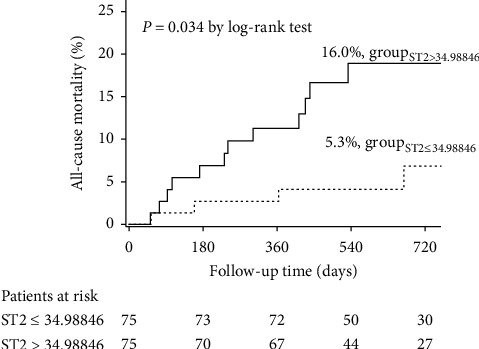
Kaplan–Meier curves for all-cause mortality according to the median levels of sST2.

The incidence of all-cause mortality was significantly increased in patients with sST2 levels above 34.98846 ng/ml (16.00% vs. 5.33%, respectively, log-rank *P* = 0.034) (Figure 1). When considering the model (age, gender, device implantation, prevention of sudden death, LVEDD, LVEF, WBC and CLBBB, hsTNT, etiology, and eGFR) and the model combined with NT-proBNP in the Cox multivariable regression, the risk of all-cause death was increased by 2.5% (HR: 1.025, [95% CI, 1.007–1.043]) and 1.9% (HR: 1.019, [95% CI, 1.000–1.037]), respectively, per ng/ml of sST2 (Table 2).

**Figure 2:**
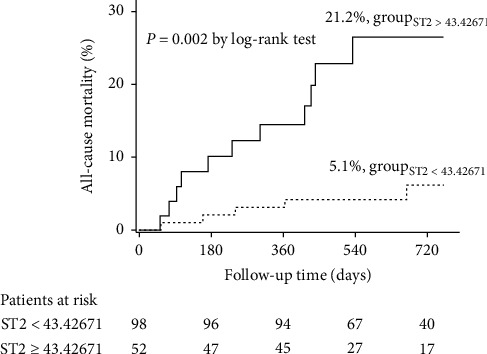
Kaplan–Meier curves for all-cause mortality according to the optimal cutoff of sST2.

The best sST2 cutoff for predicting all-cause death was 43.42671 ng/ml (area under the curve: 0.72, sensitive: 0.69, specificity: 0.69). This cutoff allowed good risk stratification in the Kaplan–Meier analysis. Compared with patients showing sST2 levels below 43.42671 ng/ml, the risk of all-cause mortality was higher in the patients with levels above the threshold (5.1% vs. 21.2%, respectively, log-rank *P* = 0.002) (Figure 2). Meanwhile, sST2 level ≥43.42671 ng/ml was an independent predictor of all-cause mortality (HR: 3.30 [95% CI 1.02–10.67]). Age (HR: 1.06 [95% CI 1.01-1.12]) and increased NT-proBNP per 100 (HR: 1.02 [95% CI 1.01–1.03]) were also associated with all-cause mortality in ICD patients (Table 3).

## 4. Discussion

In the present study, we focused on patients with severe heart failure who were suitable and implanted with ICD to further decrease mortality from heart failure. In long-term observation after ICD therapy, we found that the levels of sST2 43.43 ng/ml could predict the all-cause mortality efficiently. NT-proBNP, sST2, and age have also been described as independent predictors for all-cause mortality in HF with ICD.

Our results are consistent with the study by Hicham et al. reporting that sST2 and NT-proBNP are promising biomarkers for identifying patients with little potential to gain a significant survival benefit from ICD therapy [25]. Both these observations present the fact that ICD therapy is associated with significant morbidity. Although most patients implanted with an ICD based on current guidelines will receive a lifesaving device therapy, many patients with advanced heart failure will not have their life meaningfully prolonged by ICD therapy. Determining the cost-effectiveness of the therapy for patients implanted with an ICD based on current guidelines is still a challenge.

NPs are the major biomarkers used for heart failure including both BNP and NT-proBNP, which have become a part of routine care for HF diagnoses in both acute and ambulatory settings. Although the prognostic value of NPs is well established, their adoption by clinicians has been irregular because NT-proBNP is affected by many factors such as gender, age, BMI, and renal function [9–11, 29]. Based on these limitations, more novel biomarkers are being studied to assess HF conditions, including sST2, GDF15, and IL-6. In the present study, we chose sST2 as a novel biomarker that contributes to the formation of myocardial fibrosis and ventricular remodeling [14, 21–23, 30–33]. Since its discovery in 1989, increasing studies have confirmed that cardiomyocytes and cardiac fibroblasts induce release of both lST2 and sST2 when subjected to mechanical stretching in HF patients and in animal models. The predictive value of sST2 among HF patients has been revealed in several studies [12–25]. Interestingly, our best cutoff point of sST2 as a long-term mortality predictor in this study was 43.42671 ng/ml, which is greater than the traditional cutoff level of 35 ng/ml, possibly due to the additional protection of ICD therapy along with optimal medication, in these high-risk heart failure patients. All-cause mortality risk was at least more than three times in patients with sST2 levels at 43.42671 ng/ml than in those with levels <43.42671 ng/ml (HR: 3.30; 21.2% vs 5.1%, log-rank *P* = 0.002) among heart failure patients with ICD. The results imply that the baseline sST2 has significant prognostic value in HF patients with ICD and that patients with sST2 levels greater than 43.42671 ng/ml should be monitored carefully even after ICD implantation.

Overall, we explored the predictive effect of sST2 on all-cause mortality in patients with heart failure and EF <35% and implanted with cardiac devices. For the first time, we proposed the threshold of sST2 ≥43.42671 ng/ml as a good distinguishing cutoff value to predict all-cause mortality.

Based on the above research results, we will design a large-sample, multicenter study to further determine the predictive effect of sST2 >43 in all-cause mortality in patients with EF <35% ICD implantation in the next study. Moreover, for patients with sST2 >43 obtained in this study, we will further give more intensive medical therapies that can improve the short-term or long-term prognosis of patients.

### 4.1. Study Limitations

First, our study sample is small, and the number of deaths is low. The results were derived from a single-center study with a small patient cohort, and the enrollment period was only 20 months. This is probably because all the patients were eligible and were implanted with a cardiac device. Larger cohorts are required to assess the sensitivity and specificity of prognostic performance measurements. Secondly, the follow-up was not long enough. All patients were followed up for at least 1 year; the longest follow-up was more than 2 years. In all, 100% of real-world ICD patients were followed up in this study. Only 16 patients died in our study among 150 HF patients with ICD, and the death rate in our study (10.67%) was similar to the 1-year mortality rate in the ICD arm of MADIT-II (9%). Third, although all patients had received adequate management based on guideline-recommended therapy, the patient management was at the discretion for the different treating physician which still was a limitation. Fourth, this study did not include an in-depth analysis of pathophysiological mechanisms and signaling pathways.

## 5. Conclusion

The level of sST2 was found to be associated with the risk of all-cause mortality. The sST2 threshold of 43.43 ng/ml had a good discrimination performance to predict all-cause mortality in severe heart failure patients recommended for ICD implantation.

## Figures and Tables

**Table 1:** Baseline characteristics and medical management according to the median levels of sST2.

	All patients (*N* = 150)	ST ≤ 34.98846 (ng/ml) (*N* = 75)	ST2 > 34.98846 (ng/ml) (*N* = 75)	*P* value
Age, yrs	61.63 ± 11.14	61.39 ± 10.27	61.87 ± 12.02	0.79
Men	113 (75.33%)	55 (73.33%)	58 (77.33%)	0.57
BMI, kg/m^2^	24.23 ± 3.21	24.57 ± 3.08	23.88 ± 3.33	0.19
NYHA function classes				0.38
I and II	47 (31.33%)	21 (28.00%)	26 (34.67%)	—
III and IV	103 (68.67%)	54 (72.00%)	49 (65.33%)	—
Hypertension	53 (35.33%)	31 (41.33%)	22 (29.33%)	0.12
Diabetes	42 (28.00%)	21 (28.00%)	21 (28.00%)	1.00
CLBBB	62 (41.33%)	39 (52.00%)	23 (30.67%)	0.01
Ischemic etiology	70 (46.67%)	36 (48.00%)	34 (45.33%)	0.74
Dilated cardiomyopathy	73 (48.67%)	37 (49.33%)	36 (48.00%)	0.87
LA, mm	44.00 (40.00–49.00)	43.00 (40.00–48.00)	45.00 (40.00–51.00)	0.15
LVEDD, mm	66.00 (62.00–74.00)	66.00 (62.00–74.00)	67.00 (62.00–76.00)	0.82
LVEF (%)	29.41 ± 4.69	30.29 ± 4.50	28.53 ± 4.74	0.02
WBC, 10^9^/L	7.72 ± 2.34	7.34 ± 1.81	8.10 ± 2.73	0.05
Device				0.02
ICD	80 (53.33%)	33 (44.00%)	47 (62.67%)	
CRT-D	70 (46.67%)	42 (56.00%)	28 (37.33%)	
SCD prevention				0.003
Primary	110 (73.33%)	63 (84.00%)	47 (62.67%)	
Secondary	40 (26.67%)	12 (16.00%)	28 (37.33%)	
eGFR, ml/min/1.73 m^2^	78.37 ± 31.95	81.08 ± 28.49	75.67 ± 35.06	0.30
HsTNT, ng/ml	0.03 (0.01–0.05)	0.02 (0.01–0.05)	0.03 (0.02–0.05)	0.88
NT-proBNP, pg/ml	1546.50 (750.20–3119.00)	1407.00 (673.10–2967.00)	1844.00 (789.00–4004.00)	0.10
sST2, ng/ml	34.99 (26.20–47.19)	26.20 (22.18–31.10)	47.19 (42.24–62.94)	<0.0001
ACEI	77 (51.33%)	36 (48.00%)	41 (54.67%)	0.41
ARB	34 (22.67%)	21 (28.00%)	13 (17.33%)	0.12
*β*-Blocker	107 (71.33%)	50 (66.67%)	57 (76.00%)	0.21
Uretic	136 (90.67%)	68 (90.67%)	68 (90.67%)	1.00

Values are mean ± SD, median (interquartile range), or No. (%). BMI, body mass index; NYHA, New York Heart Association; CLBBB, complete left bundle branch block; LVEF, lower left ventricular ejection fraction; CRT-D, cardiac resynchronization defibrillator; ICD, implantable cardioverter defibrillator; SCD, sudden cardiac death; eGFR, estimated glomerular filtration rate; hsTnT, high-sensitivity troponin T; NT-proBNP, terminal protype natriuretic peptide; sST2, soluble suppression of tumorigenesis-2; ACEI, angiotensin-converting enzyme inhibitor; ARB, angiotensin receptor blocker.

**Table 2:** Prognostic value of sST2 in all-cause mortality.

	Variables for adjustment	HR (95% CI)	*P* value
sST2	—	1.022 (1.011–1.034)	0.0001
sST2	Model	1.025 (1.007–1.043)	0.0060
sST2	Model + NT-proBNP	1.019 (1.000–1.037)	0.0455

HR, hazard ratio; CI, confidence interval. Other abbreviations are the same as for Table 1. The prognostic model includes age, gender, device type, SCD prevention, LVEDD, LVEF, WBC, CLBBB, ischemic cardiomyopathy, and eGFR.

**Table 3:** The independent predictors of all-cause mortality.

	Univariate regression		Multivariate regression	
	Unadjusted HR (95% CI)	*P* value	Adjusted HR (95% CI)	*P* value
Age	1.08 (1.02–1.13)	0.004	1.06 (1.01–1.12)	0.01
sST2 ≥43.43 ng/ml	4.66 (1.62–13.44)	0.004	3.30 (1.02–10.67)	0.046
NT-proBNP per 100 increased	1.02 (1.01–1.03)	<0.001	1.02 (1.01–1.03)	0.004

HR, hazard ratio; CI, confidence interval. Other abbreviations are the same as for Table 1.

## Data Availability

The data used to support the findings of this study are included within the article.

## Supplementary Materials

Supplementary Materials Table S1: baseline characteristics and medical management according to the patients with survival status.
