# Supplementary material for: Circulating Soluble ST2 Predicts All-Cause Mortality in Severe Heart Failure Patients with an Implantable Cardioverter Defibrillator
Source: Cardiol Res Pract. 2020 Nov 17;2020:4375651. doi: 10.1155/2020/4375651 (PMC7685860; doi:10.1155/2020/4375651)
Supplement: Supplementary Materials — Table S1: baseline characteristics and medical management according to the patients with survival status. [file 4375651.f1.docx]

Table S1. Baseline characteristics and medical management according to the patients with survival status

|  | **Survival group (N=134)** | **Death group**  **(N=16)** | **P value** |
| --- | --- | --- | --- |
| Age，yrs | 60.70±11.03 | 69.38±9.14 | 0.002956557 |
| Men | 100(74.63%) | 13(81.25%) | 0.5613 |
| BMI，kg/m2 | 24.39±3.11 | 22.82±3.79 | 0.063852144 |
| NYHA function classes |  |  | 0.5634 |
| I AND II | 43(32.09%) | 4(25.00%) |  |
| III AND IV | 91(67.91%) | 12(75.00%) |  |
| Hypertension | 49(36.57%) | 4(25.00%) | 0.3603 |
| Diabetes | 37(27.61%) | 5(31.25%) | 0.7594 |
| CLBBB | 55(41.04%) | 7(43.75%) | 0.8355 |
| Ischemic etiology | 63(47.01%) | 7(43.75%) | 0.8046 |
| Dilated cardiomyopathy | 65(48.51%) | 8(50.00%) | 0.9101 |
| LA mm | 44.00 (40.00-48.00) | 48.50 (42.50-53.00) | 0.052 |
| LVEDD mm | 66.00 (62.00-74.00) | 67.50 (63.00-74.00) | 0.64 |
| LVEF(%) | 29.63±4.71 | 27.56±4.23 | 0.094851777 |
| WBC 10^9^/L | 7.75±2.42 | 7.44±1.65 | 0.619448666 |
| Device |  |  | 0.8046 |
| CRT-D | 63(47.01%) | 7(43.75%) |  |
| ICD | 71(52.99%) | 9(56.25%) |  |
| SCD prevention |  |  | 0.4487 |
| primary | 97(72.39%) | 13(81.25%) |  |
| secondary | 37(27.61%) | 3(18.75%) |  |
| eGFR ml/min/1.73m^2^ | 80.45±31.65 | 60.97±29.95 | 0.020638898 |
| hsTNT ng/ml | 0.02(0.01-0.05) | 0.03(0.03-0.09) | 0.0246 |
| NT_proBNP pg/ml | 1417.00(673.10-2961.00) | 4100.50(2428.50-7077.00) | 0.0002 |
| ST2 ng/ml | 38.71±19.63 | 62.20±45.57 | 0.05866569 |
| ST2(median Q1,Q3) | 33.80(25.72-45.43) | 45.98(35.09-67.29) | 0.0046 |
| ACEI | 70(52.24%) | 7(43.75%) | 0.5208 |
| ARB | 34(25.37%) | 0(0.00%) | 0.022 |
| β-blocker | 94(70.15%) | 13(81.25%) | 0.3534 |
| Uretic | 121(90.30%) | 15(93.75%) | 0.6537 |

Values are mean±SD, median (interquartile range), or No. (%). Abbreviation: BMI, body mass index; NYHA, New York Heart Association; CLBBB, complete left bundle branch block; LVEF, lower left ventricular ejection fraction; CRT-D, cardiac resynchronization defibrillator; ICD, implantable cardioverter defibrillator; SCD, sudden cardiac death; eGFR, estimated glomerular filtration rate; hs TnT, high-sensitivity troponin T; NT proBNP, terminal pro-type natriuretic peptide; sST 2, soluble suppression of tumorigenesis-2; ACEI, angiotensin-converting enzyme inhibitor; ARB, angiotensin receptor blocker.
